# Acute toxicity and ecological risk assessment of 4,4’-dihydroxybenzophenone, 2,4,4’-trihydroxybenzophenone and 4-MBC in ultraviolet (UV)-filters

**DOI:** 10.1371/journal.pone.0249915

**Published:** 2021-04-08

**Authors:** Jing Han, Ze-Tian Qin, Jing Zhang, Wen-Qiang Wang, Jing-Ya Wu, Yong-Ze Lu, Li-Wei Sun

**Affiliations:** 1 School of Energy and Environment, Southeast University, Nanjing, China; 2 Taihu Lake Water Environment Engineering Research Centre (Wuxi), Southeast University, Wuxi, China; Government College University Faisalabad, Pakistan, PAKISTAN

## Abstract

Ultraviolet (UV) filters are used in cosmetics, personal care products and packaging materials to provide sun protection for human skin and other substances. Little is known about these substances, but they continue to be released into the environment. The acute toxicity of 4,4’-dihydroxybenzophenone, 2,4,4’-trihydroxybenzophenone and 4-MBC to *Chlorella vulgaris* and *Daphnia magna* were analyzed in this study. The 96 h-EC_50_ values of 4,4’-dihydroxybenzophenone, 2,4,4’-trihydroxybenzophenone and 4-MBC on *C*. *vulgaris* were 183.60, 3.50 and 0.16874 mg/L, respectively. The 48 h-LC_50_ of 4,4’-dihydroxybenzophenone, 2,4,4’-trihydroxybenzophenone and 4-MBC on *D*. *magna* were 12.50, 3.74 and 0.54445 mg/L, respectively. The toxicity of a mixture of 4,4’-dihydroxybenzophenone and 4-MBC showed addictive effect on *C*. *vulgaris*, while the toxicity of mixtures of 4,4’-dihydroxybenzophenone and 2,4,4’-trihydroxybenzophenone, 2,4,4’-trihydroxybenzophenone and 4-MBC as well as 4,4’-dihydroxybenzophenone, 2,4,4’-trihydroxybenzophenone and 4-MBC all showed antagonistic effect on *C*. *vulgaris*. The induced no-effect concentrations of 4,4’-dihydroxybenzophenone, 2,4,4’-trihydroxybenzophenone and 4-MBC by the assessment factor (AF) method were 0.0125, 0.00350 and 0.000169 mg/L, respectively.

## Introduction

Ultraviolet exposure can do harm to human skin as well as alter the properties of substances like polymer in food and other products [1]. Such character of ultraviolet prompted the widespread use of UV filters. UV filters are the main ingredient in sunscreens that provide sun protection, and are also used as UV stabilizers in hair sprays, insect repellents, plastics and rubbers because they can prevent polymer degradation or pigmentation, working either by absorbing UV radiation (known as organic absorbers) or reflecting UV radiation (known as inorganic blockers) [2]. 14 benzophenone-type UV filters (including 4,4’-dihydroxybenzophenone and 2,4,4’-trihydroxybenzophenone) as well as camphor derivatives (including 4-methylbenzylidene camphor, i.e. 4-MBC) are among the organic absorbers that are commonly used as UV filers [3]. Hydroxylated benzophenones are not only used in a variety of commercial products like consmetics and plastics, but also metabolites of benzophenone and its derivatives in living organisms [4]. These chemicals enter the aquatic environment via wastewater discharge (“indirect input”) as well as recreational activities in rivers and lakes (“direct input”). It is reported that wastewater treatment plants are not fully capable of removing UV filters from sewage [5], so considerable amounts of such chemicals enter the environment through wastewater discharge. UV filters, especially benzophenones, have been frequently detected in the aquatic environment.

In a study in Taiwan, BP-3 (benzophenone-3), BP-1 (benzophenone-1) and BP-8 (benzophenone-8) were detected in wastewater effluents, with their concentration ranging from 7.7 to 21.4 ng/L [6]. A study measured eight UV filters in water and sediment samples from 4 wastewater treatment plant in Japan, where BP-3 and EHMC (ethylhexyl methoxy cinnamate) were both detected [7]. In a recreational lake in Switzerland, 4-MBC, EHMC, OC (octocrylene) and BP-3 were detected [8]. A review elaborated that the highest l concentration of BP-3 reached 125 ng/L in freshwater and 577.5 ng/L in seawater [2]. Seawater samples taken from Liguria, Italy were proven to contain BP-3 and EHMC, the concentrations ranged from 25–118 ng/L [9]. Due to the high lipophilicity of most UV filters, they are frequently detected as well as in soil or sediments. It has been reported that the concentrations of BP-1, BP-3 and BP-8 in soil and sediments could reach up to 0.61, 27 and 4.17 ng/g dw (dry weight) respectively [10]. Since most UV filters are photostable and lipophilic, they are hard to be degraded and easily bioaccumulated in the environment [11]. It’s reported that the EHMC concentration in macroinvertebrate and fish was 337 ng/g, but the concentration increased to 701 ng/g in cormorants [12] which prey on fish. Fent et al. studied the acute toxicity of BP-3, BP-4, 4-MBC and EHMC to *D*. *magna*, and found the 48 h-LC_50_ values were 50 mg/L, 1.9 mg/L, 0.56 mg/L and 0.59 mg/L respectively [13]. BP-3, 4-MBC, OMC (octyl methoxycinnamate) and OD-PABA (4-amino-,2-ethylhexyl ester) were reported to have endocrine-disrupting capacities, while BP-2 (benzophenone-2) was found to affect the segmentation process, blood circulation, lipid metabolism, and facial formation in zebrafish embryos [14]. In our previous study, the 96 h-EC_50_ values of BP, BP-1, BP-2, BP-3, BP-4 on *C*. *vulgaris* were reported as 6.86, 29.70, 190.67, 2.98 and 201.00 mg/L, respectively, and the 48 h-LC_50_ of these chemicals on *D*. *magna* were 7.63, 17.23, 52.81, 1.09, 47.47 mg/L [15, 16].

**Fig 1:**
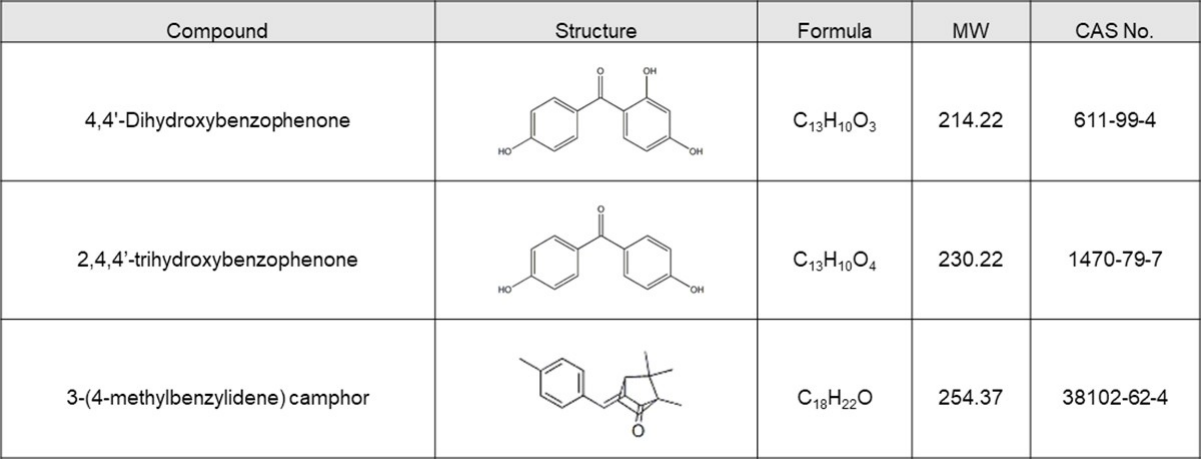
Structures of tested chemicals.

However, the toxicity data of UV filters are still scarce, and these chemicals have not been included in any environmental criteria or discharge standards. Despite their relatively low concentration, UV filter chemicals are still worth attention due to their adverse effect both *in vivo* and *in vitro* as well as their risk of bioaccumulation. In this study, we focused on the acute toxicity of 4,4’-dihydroxybenzophenone, 2,4,4’-trihydroxybenzophenone and 4-MBC (Fig 1), to aquatic biota and their environmental risks.

The aim of this study was to investigate the acute ecological toxicity of 4,4’-dihydroxybenzophenone, 2,4,4’-trihydroxybenzophenone and 4-MBC to aquatic organisms, thereby to evaluate their potential environmental risk. The acute toxicity of the three chemicals, both separated and mixed were tested. Two different freshwater biota species: *Chlorella vulgaris* and *Daphnia magna*, were employed in the acute toxicity test for separated chemicals, while *C*. *vulgaris* was used in the mixed tests.

Based on the result of acute toxicity test, the toxicity grades of the three chemicals were determined according to the acute toxicity test classification criteria. The degree and the types of mixed effects were obtained in mixed tests. The predicted no-effect concentration (PNEC) was predicted to evaluate their ecological risks. The results of the study will support to make environmental criteria.

All the experiments were conducted in accordance with Chinese National Standards: GB/T 21805–2008 (*C*. *vulgaris*) [17] and GB/T 16125–2012 (*D*. *magna*) [18], as well as the Analytical Methods for Water and Wastewater [19].

## Materials and methods

### Chemicals and reagents

4,4’-dihydroxybenzophenone, 2,4,4’-trihydroxybenzophenone and 4-MBC at 99% purity were obtained from Aladdin Industrial Corporation (Shanghai, China). Dimethyl sulfoxide (Sinopharm Chemical Reagent Co., Ltd., Shanghai, China) at analytical purity grade was used as a cosolvent.

### Experimental biota for toxicity testing

*Chlorella vulgaris* (FACHB-8) was obtained from the Freshwater Algae Culture Collection of the Institute of Hydrobiology, Chinese Academy of Science (Wuhan, China). The Blue-green algae medium (BG11) was used for the culturing of *C*. *vulgaris*. All the experiments were carried out under the same condition: 2300–2800 Lux, 25±5°C, and a 12-h light: dark cycle. Algae cells in the logarithmic growth phase were used in the experiment. The algae density-absorbance curve was plotted based on the algae absorbance and the *C*. *vulgaris* cell density. A series density of algae solution was prepared, the absorbance was measured at 680 nm with a 7600 UV-visible spectrophotometer, and then the corresponding cell densities of *C*. *vulgaris* were counted under the optical microscope (*Olympus*, ckx 41) on a hemocytometer (*Marienfeld*).

All samples were counted more than three times in which the errors between each sample was less than 10%. In the subsequent experiments, the algae densities were calculated by measuring the absorbance based on the absorbance-density curve.

*D*. *magna* were obtained from the Institute of Hydrobiology, Chinese Academy of Science (Wuhan, China), cultured in aerated tap water and fed by the *C*. *vulgaris*. All the experiments were conducted under the condition: 2300–2800 Lux, 23±2°C, pH = 6.8–7.2, DO = 8.0–8.6mg/L, and a 12-h light: dark cycle. A test for their sensitivity to potassium dichromate was conducted before the toxicity tests. *D*. *magna* between 6-24h old were used in the experiment.

### Classification criteria for acute toxicity test

The classification criteria for acute toxicity of 4,4’-dihydroxybenzophenone, 2,4,4’-trihydroxybenzophenone and 4-MBC were listed as follows: 1) acute toxicity test classification criteria for algae (Table 1) and 2) acute toxicity test classification criteria for Daphnia (Table 2). The classification criteria were excerpted from Analytical Methods for Water and Wastewater [19].

**Table 1:** Acute toxicity test classification criteria for algae.

96 h-EC_50_ (mg/L)	Toxicity Level
<1	Very high-level
1–10	High-level
10–100	Medium-level
>100	Low-level

**Table 2:** Acute toxicity test classification criteria for Daphnia.

48 h-LC_50_ (mg/L)	Toxicity Level
<1	Very high-level
1–10	High-level
10–100	Medium-level
>100	Low-level

### Experimental design

#### Acute toxicity experiment (96h) with *Chlorella vulgaris*

Stock solutions of 4,4’-dihydroxybenzophenone, 2,4,4’-trihydroxybenzophenone and 4-MBC were prepared using cosolvent, Dimethyl sulfoxide (DSMO). The exposed concentrations were decided according to the results of a pre-experiment. The concentrations were set as 160, 170, 180, 190 and 200 mg/L for 4,4’-dihydroxybenzophenone, 1, 2, 3, 4 and 5 mg/L for 2,4,4’-trihydroxybenzophenone and 0.100, 0.130, 0.160, 0.180 and 0.200 mg/L for 4-MBC. The initial concentration of *C*. *vulgaris* was prepared at 5.24×10^3^ cells/mL to 5.73×10^4^ cells/mL. The experiment lasted for 96 h. A blank group was included and three parallel samples of each group were set. During the test period, the absorbance was measured every 24h and the density of *C*. *vulgaris* was calculated to evaluate the inhibition effects on algae growth. In the blank group, *C*. *vulgaris* cells increased over 16 fold, This result met the quality control requirements for *C*. *vulgaris* acute toxicity tests [17].

#### Acute toxicity experiment (48h) with *Daphnia magna*

The concentrations of experimental solution, decided according to the results of a pre-experiment, were set as 5, 10, 15, 20 and 25 mg/L for 4,4’-dihydroxybenzophenone, 0.1, 0.5, 1, 5 and 10 mg/L for 2,4,4’-trihydroxybenzophenone and 0.200, 0.400, 0.600, 0.800 and 1.000 mg/L for 4-MBC. Dimethyl sulfoxide (DSMO) was used as cosolvent. The 40 mL solution were prepared in 100-mL glass beakers. Ten *D*. *magna* were added to each beaker. The test lasted for 48 h, during which no feedstuffs were offered. Dead individuals were removed promptly so as not to pollute the water. A blank control group was included and three parallel samples of each group were tested. The numbers of dead *D*. *magna* were recorded every 24 h to evaluate the lethal effect of 4,4’-dihydroxybenzophenone, 2,4,4’-trihydroxybenzophenone and 4-MBC on *D*. *magna*. The sensitivity of *D*. *magna* was tested with potassium dichromate before the experiment. The results met the quality control requirements for *D*. *magna* acute toxicity tests [18].

#### The mixed toxicity experiment

The toxicity of mixtures of 4,4’-dihydroxybenzophenone, 2,4,4’-trihydroxybenzophenone and 4-MBC were tested using the same method as for the separated test. The initial concentrations of the tested chemicals, which were set as 100% mixture stock solution, were decided based on the EC_50_ values derived from separated tests.

A series of diluted concentrations of the mixtures were prepared, i.e. 50%, 40%, 30%, 20% and 10%. Toxicities of the mixtures were decided by calculating the toxicity units (TU) with the formula listed below:

$${TU}_{i}=\frac{C_{i}}{{EC}_{50i}}$$ (1)

where C_i_ is the concentration of i in tested solution at the EC_50_ of the mixture.

The total TU value was the sum of the *TU*_*i*_ values of individual compounds. A total TU value greater than 1 indicated the toxicity be antagonistic, and a total TU value equal to 1 indicated the toxicity be an addictive effect, and a total TU value less than 1 indicated the toxicity be synergistic.

### Statistical analysis

OriginPro 2021 software (OriginLab, Northampton, MA, USA) was applied to draw graphs depicting the results of experiment. IBM SPSS version 21 software (IBM Corp., Armonk, NY, USA) was used to calculate the 96-h EC_50_ value of *C*. *vulgaris* and the 48-h LC_50_ value of *D*. *magna*.

All relevant data in this research are within the manuscript and its (S1 File).

### Ecological risk assessment

PNEC values were calculated according to the acute toxicity data of present study and those previously recorded in the dominant database. The assessment factor (AF) was employed to determine the PNEC value, where OECD recommended UF value 1000 was applied.

## Results

### Acute toxicity of 4,4’-dihydroxybenzophenone, 2,4,4’-trihydroxybenzophenone and 4-MBC to aquatic biota

#### *C*. *vulgaris* growth inhibition test of 4,4’-dihydroxybenzophenone, 2,4,4’-trihydroxybenzophenone and 4-MBC

It has been proven in our previous study that dimethyl sulfoxide, used as the cosolvent in this study, has no significant effect on *C*. *vulgaris* and *D*. *magna*, and therefore did not interfere with the experimental results [15].

**Fig 2:**
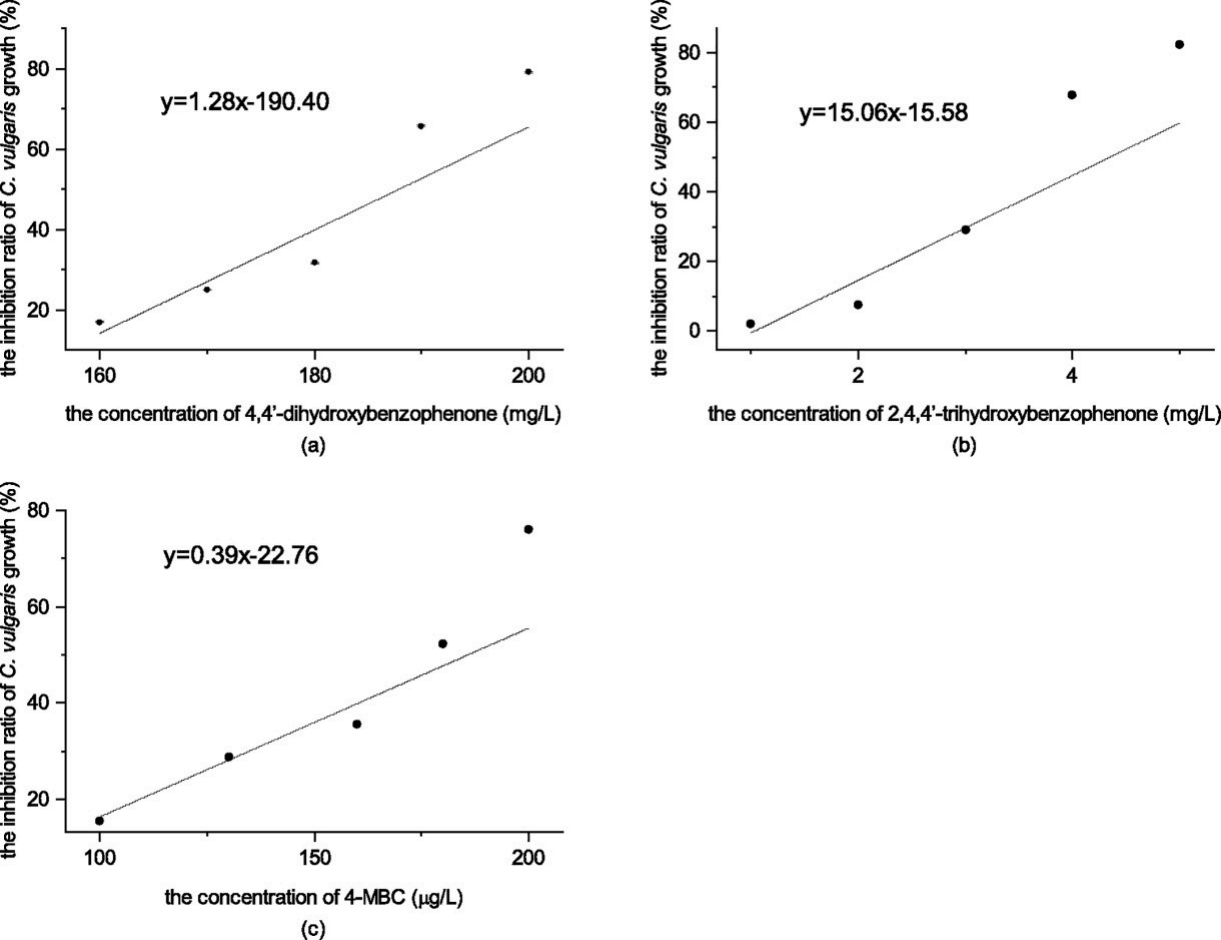
(a) The inhibition ratio of *C*. *vulgaris* growth of 4,4’-dihydroxybenzophenone; (b) The inhibition ratio of *C*. *vulgaris* growth of 2,4,4’-trihydroxybenzophenone; (c) The inhibition ratio of *C*. *vulgaris* growth of 4-MBC.

The growth inhibition of *C*. *vulgaris* was positively correlated with the concentration of the three chemicals within the set experimental ranges. Fig 2 shows the relationship between the growth inhibition rate of *C*. *vulgaris* and the concentration of three tested chemicals as:

4,4’-dihydroxybenzophenone:

$$y=1.27x-190.4,R^{2}=0.8550$$ (2)

2,4,4’-trihydroxybenzophenone:

$$y=15.56x-15.58,R^{2}=0.7877$$ (3)

4-MBC:

$$y=0.39x-22.76,R^{2}=0.8960$$ (4)

where *x* stands for the concentrations of tested chemicals and *y* stands for the inhibition ratios on *C*. *vulgaris* growth (%).

According to the probit analysis conducted with SPSS version 21 software, the 96 h-EC_50_ value of 4,4’-dihydroxybenzophenone, 2,4,4’-trihydroxybenzophenone and 4-MBC to *C*. *vulgaris* was 183.60 mg/L (95% confidence interval [CI] = 168.42–196.74 mg/L), 3.50 mg/L (95% confidence interval [CI] = 3.12–4.63 mg/L), and 0.16874 mg/L (95% confidence interval [CI] = 0.14539–0.18422 mg/L) respectively. By referring to the acute toxicity test classification criteria (Table 1) [19], the toxicity level of 4,4’-dihydroxybenzophenone was low-level, while 2,4,4’-trihydroxybenzophenone was high-level and 4-MBC was very high-level.

#### Acute toxicity of 4,4’-dihydroxybenzophenone, 2,4,4’-trihydroxybenzophenone and 4-MBC to *D*. *magna*

**Fig 3:**
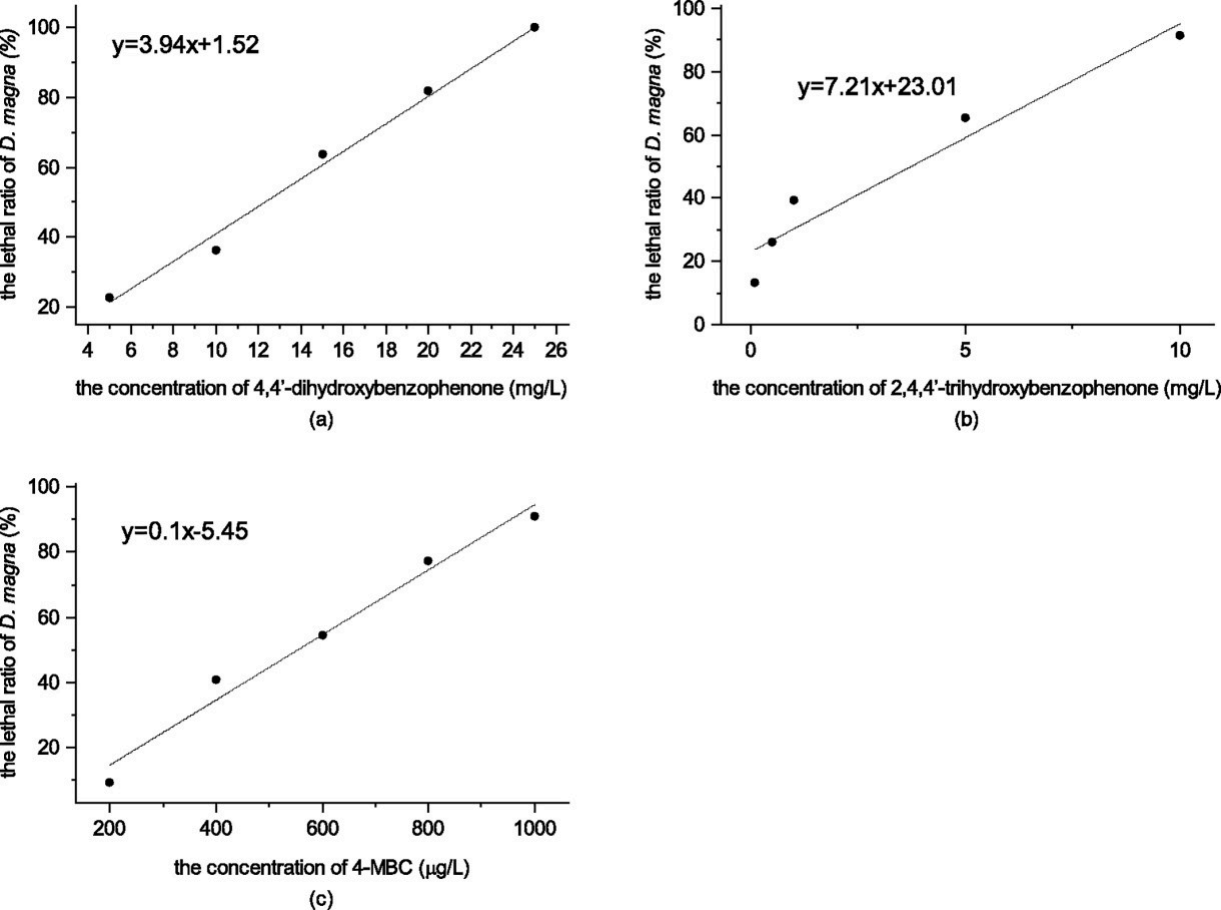
4,4’-dihydroxybenzophenone; (b) The lethal ratio of *D*. *magna* of 2,4,4’-trihydroxybenzophenone; (c) The lethal ratio of *D*. *magna* of 4-MBC.

Fig 3 shows the mortality of *D*. *magna* by the three chemicals within the experimental range, the effect-dose relationship was liner, as determined with the linear regression equation listed below:

4,4’-dihydroxybenzophenone:

$$y=2.94x+1.52,R^{2}=0.9971$$ (5)

2,4,4’-trihydroxybenzophenone:

$$y=7.21x+23.01,R^{2}=0.9376$$ (6)

4-MBC:

$$y=0.1x-5.45,R^{2}=0.9778$$ (7)

where *x* stands for the concentrations of tested chemicals and *y* stands for the mortalities of *D*. *magna* (%).

According to the probit analysis conducted with SPSS version 21 software, the 48 h-LC_50_ value of 4,4’-dihydroxybenzophenone, 2,4,4’-trihydroxybenzophenone and 4-MBC to *D*. *magna* was 12.50 mg/L (95% confidence interval [CI] = 10.27–14.95 mg/L), 3.74 mg/L (95% confidence interval [CI] = 2.86–4.21 mg/L) and 0.54 mg/L (95% confidence interval [CI] = 0.51027–0.62352 mg/L) respectively. By referring to the acute toxicity classification criteria (Table 2) [19], the toxicity of 4,4’-dihydroxybenzophenone to *D*. *magna* was determined as medium-level, while 2,4,4’-trihydroxybenzophenone was high-level and 4-MBC was very high-level.

The separate acute toxicity results are summarised in Table 3.

**Table 3:** Acute toxicity of 4,4’-dihydroxybenzophenone, 2,4,4’-trihydroxybenzophenone and 4-MBC.

	96 h-EC_50_ on *C*. *vulgaris (mg/L)*	The toxicity level on *C*. *vulgaris*	48 h-LC_50_ on *D*. *magna (mg/L)*	The toxicity level on *D*. *magna*
4,4’-dihydroxybenzophenone	183.60	Low-level	12.50	Medium-level
2,4,4’-trihydroxybenzophenone	3.50	High-level	3.74	High-level
4-MBC	0.16874	Very high-level	0.54445	Very high-level

### Acute toxicity of a mixture of 4,4’-dihydroxybenzophenone and 2,4,4’-trihydroxybenzophenone to *Chlorella vulgaris*

**Fig 4:**
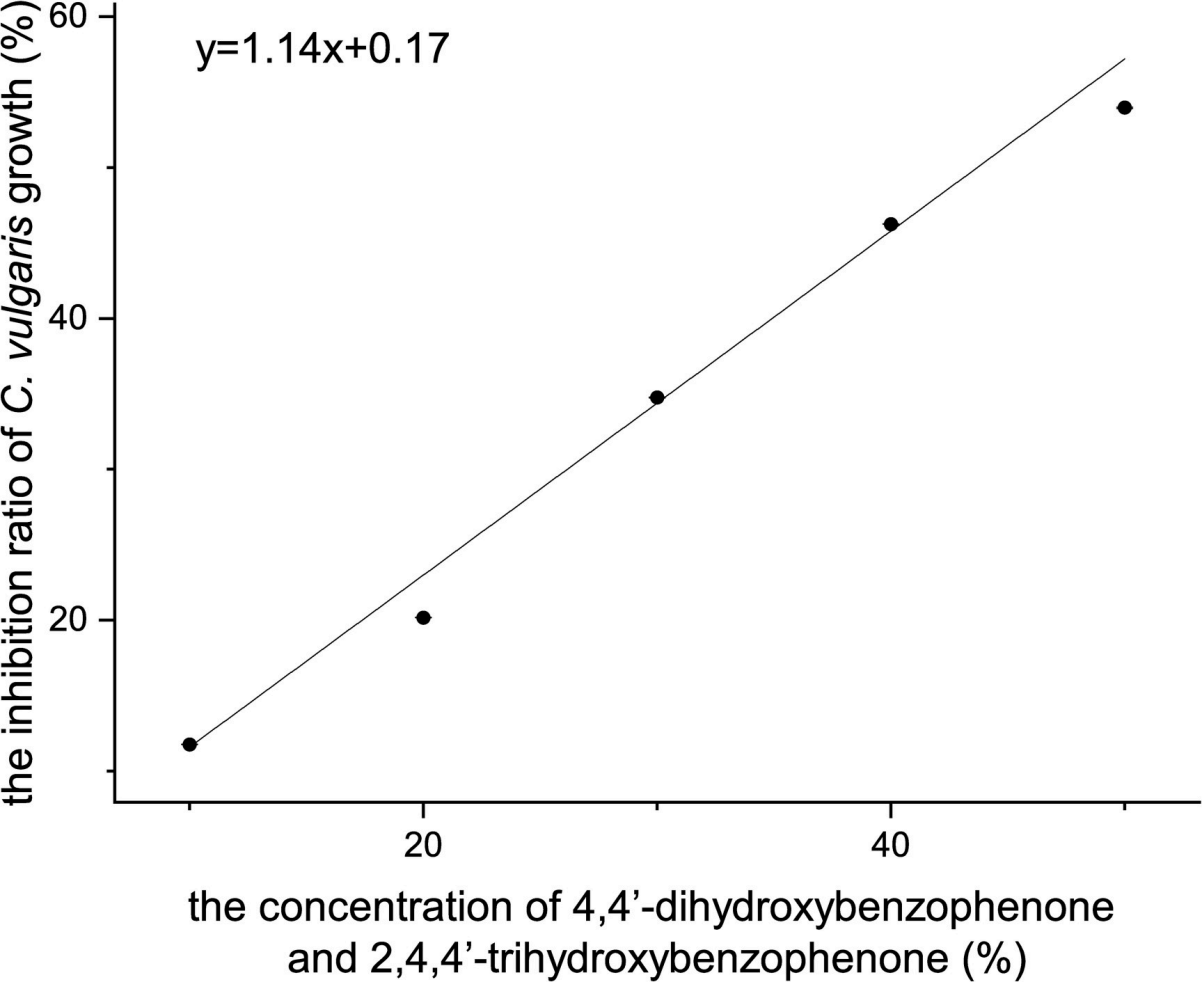
The inhibition ratio of *C*. *vulgaris* growth of a mixture of 4,4’-dihydroxybenzophenone and 2,4,4’-trihydroxybenzophenone.

Fig 4 shows that the relationship between mixture concentration and the inhibition ratio on *C*. *vulgaris* growth was linear and can be depicted as the following linear regression formula:

$$y=1.14x+0.17,R^{2}=0.9963$$ (8)

where *x* stands for the mixture concentration and *y* stands for the inhibition ratio on *C*. *vulgaris* growth (%).

The 96 h-EC_50_ value of the mixture was 45.72% (95% confidence interval [CI] = 39.24–57.18%) according to the probit analysis conducted with SPSS version 21 software, in which the concentration of 4,4’-dihydroxybenzophenone was at 90.06 mg/L and the concentration of 2,4,4’-trihydroxybenzophenone was at 5.50 mg/L. According to the result, the calculated total TU value was greater than 1, indicating that the toxicity of 4,4’-dihydroxybenzophenone and 2,4,4’-trihydroxybenzophenone mixture on *C*. *vulgaris* was antagonistic.

### Acute toxicity of a mixture of 4,4’-dihydroxybenzophenone and 4-MBC to *C*. *vulgaris*

**Fig 5:**
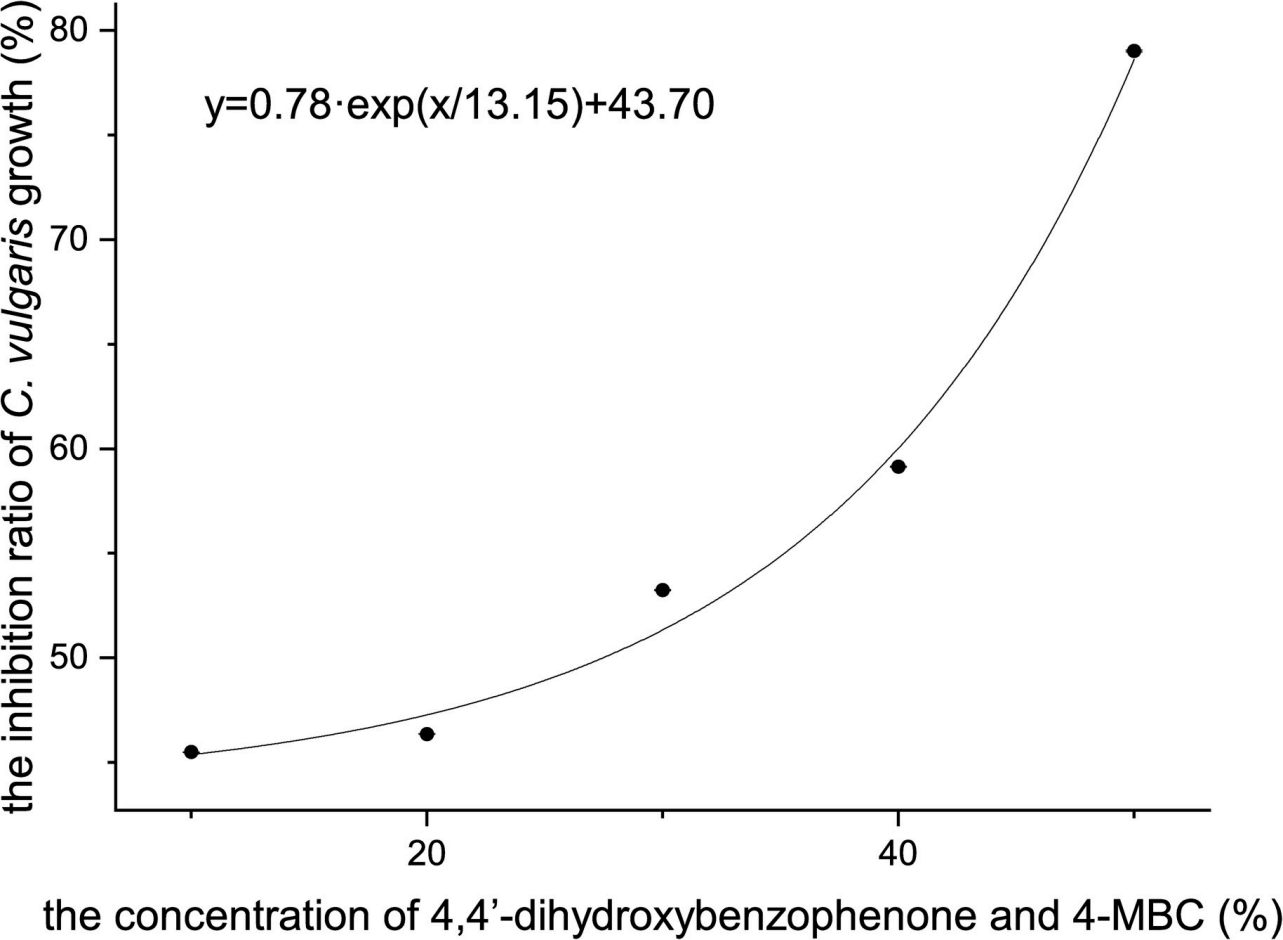
The inhibition ratio of *C*. *vulgaris* growth of a mixture of 4,4’-dihydroxybenzophenone and 4-MBC.

As is shown in Fig 5, the inhibition ratio of *C*. *vulgaris* growth showed a positive correlation with the concentration of mixture, and their relationship can be depicted with the following formula:

$$y=0.78e^{x/13.15}+43.70,R^{2}=0.9759$$ (9)

where *x* stands for the mixture concentration and *y* stands for the inhibition ratio on *C*. *vulgaris* growth (%).

According to the probit analysis conducted with SPSS version 21 software, the 96 h-EC_50_ value of the mixture was 41.97%, in which the concentration of 4,4’-dihydroxybenzophenone was at 93.94 mg/L and the concentration of 4-MBC was at 0.08677 mg/L. According to the result, the calculated total TU value was approximately equal to 1, and the toxicity of the mixture of 4,4’-dihydroxybenzophenone and 4-MBC was determined to have an addictive effect.

### Acute toxicity of a mixture of 2,4,4’-trihydroxybenzophenone and 4-MBC to *C*. *vulgaris*

**Fig 6:**
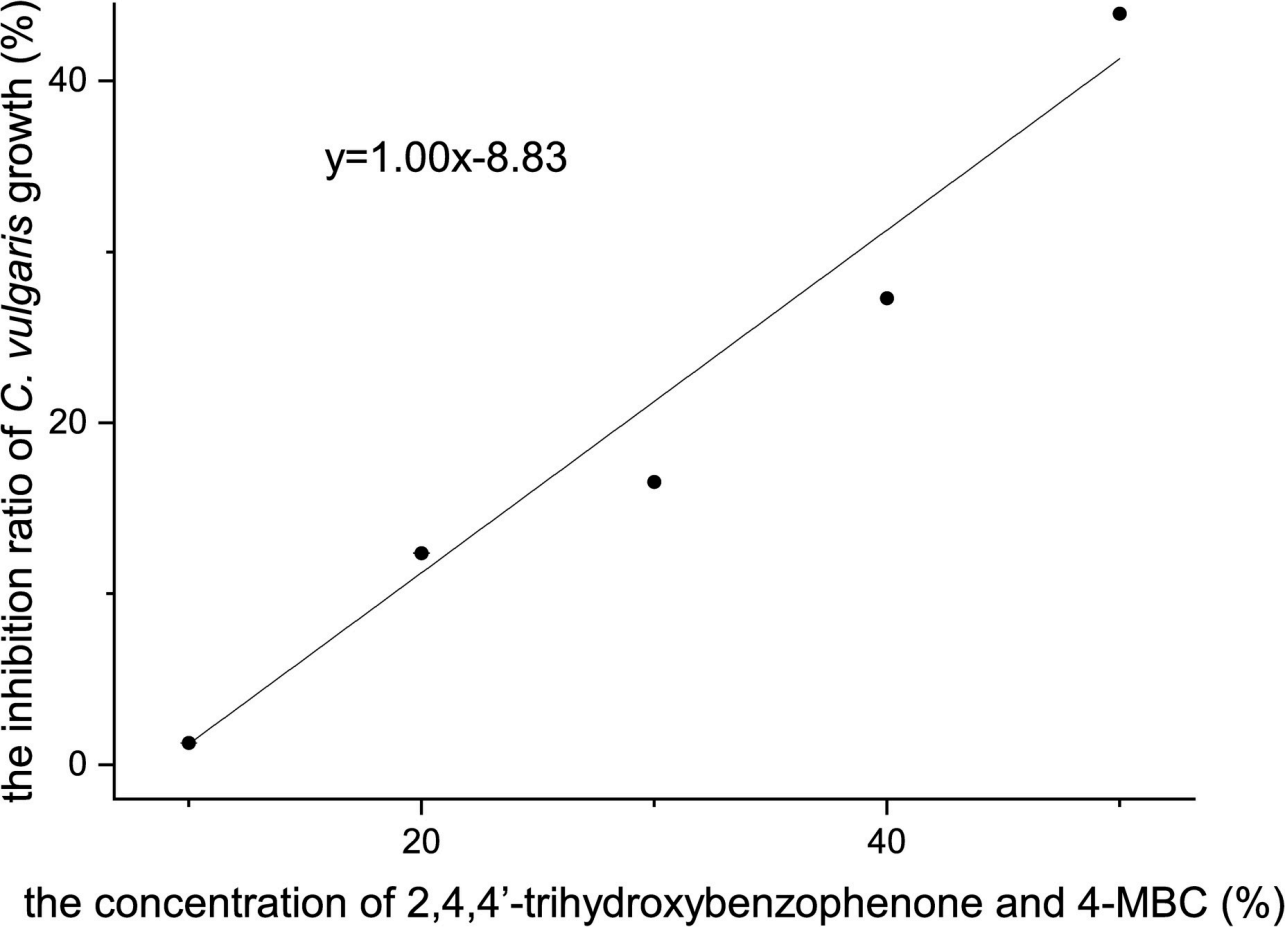
The inhibition ratio of *C*. *vulgaris* growth of a mixture of 2,4,4’-trihydroxybenzophenone and 4-MBC.

As is shown in Fig 6, the inhibition ratio of *C*. *vulgaris* growth was positively correlated with the concentration of the mixture, and their relation can be depicted with the following formula:

$$y=1.002x-9.8,R^{2}=0.9849$$ (10)

where *x* is the concentration of the mixture (%) and *y* is the inhibition ratio of *C*. *vulgaris* growth (%).

The 96 h-EC_50_ was 58.85% (95% confidence interval [CI] = 50.78% - 74.81%) according to the probit analysis conducted with SPSS version 21 software. Correspondingly, the concentrations of 2,4,4’-trihydroxybenzophenone and 4-MBC in the mixture were 5.968 and 0.16128 mg/L, respectively. According to the result, a total TU value greater than 1 was derived, indicating that 2,4,4’-trihydroxybenzophenone and 4-MBC have an antagonistic effect.

### Acute toxicity of a mixture of 4,4’-dihydroxybenzophenone, 2,4,4’-trihydroxybenzophenone and 4-MBC to *C*. *vulgaris*

**Fig 7:**
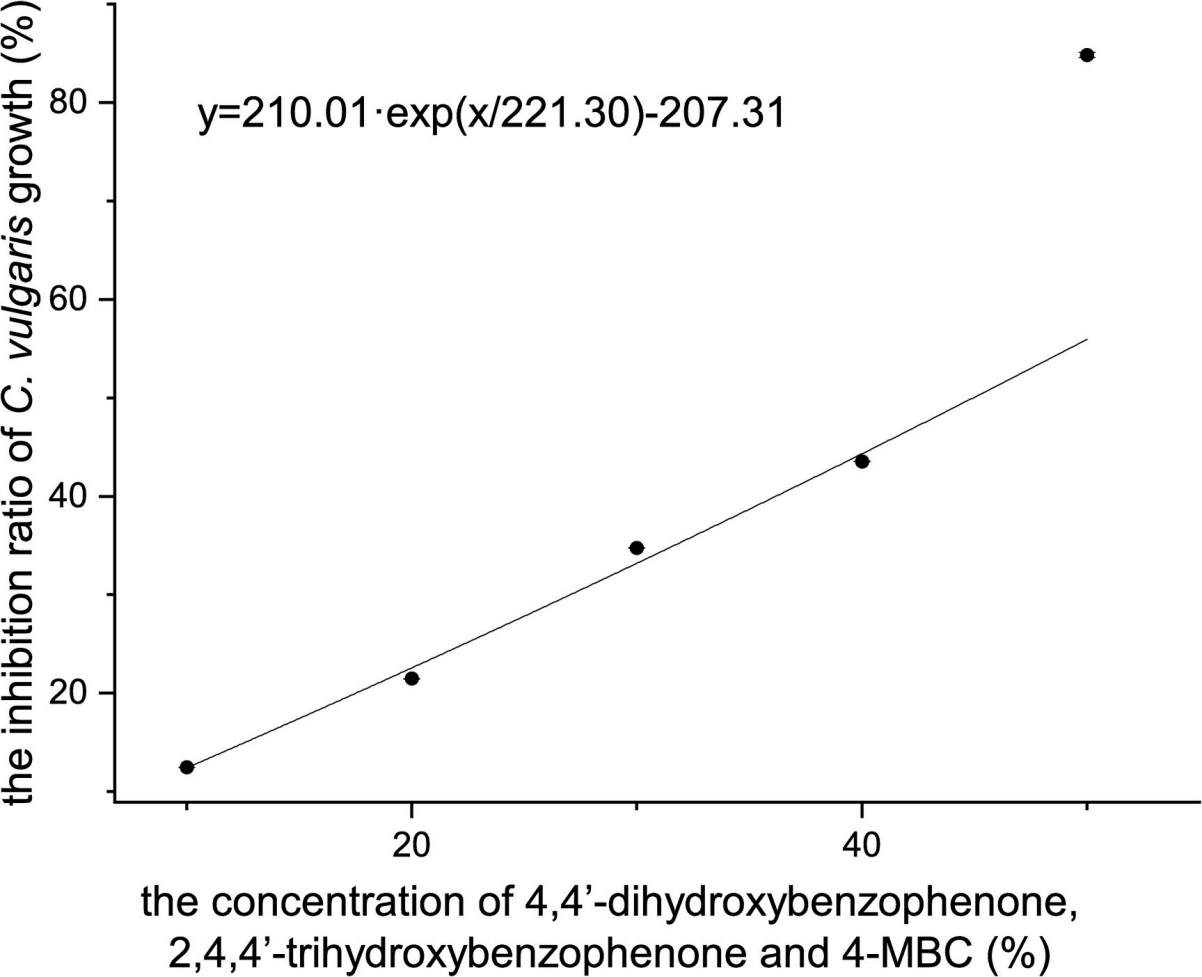
The inhibition ratio of *C*. *vulgaris* growth of a mixture of 4,4’-dihydroxybenzophenone, 2,4,4’-trihydroxybenzophenone and 4-MBC.

As is shown in Fig 7, the inhibition ratio of *C*. *vulgaris* growth was positively correlated with the concentration of the mixture. Their relation can be described with the following formula:

$$y=210.01e^{x/221.30}-207.31,R^{2}=0.9926$$ (11)

where *x* is the concentration of the mixture and *y* is the inhibition ratio of *C*. *vulgaris* growth.

The 96 h-EC_50_ of the mixture to *C*. *vulgaris* was 40.31%, as is derived from probit analysis conducted with SPSS version 21 software, in which case the concentrations of 4,4’-dihydroxybenzophenone, 2,4,4’-trihydroxybenzophenone and 4-MBC were 80.62, 4.03 and 0.16124 mg/L, respectively. The calculated total TU value was greater than one, indicating that the toxicity of 4,4’-dihydroxybenzophenone, 2,4,4’-trihydroxybenzophenone and 4-MBC mixture was antagonistic.

### Assessment of the ecological risk of 4,4’-dihydroxybenzophenone, 2,4,4’-trihydroxybenzophenone and 4-MBC

The AF method was used to determine the PNEC in present study. According to OECD recommendation, a UF value of 1000 was selected. For 4,4’-dihydroxybenzophenone, the PNEC values derived was 0.0125 mg/L (UF 1000). For 2,4,4’-trihydroxybenzophenone, the PNEC values derived was 0.0035 mg/L (UF 1000). For 4-MBC, the PNEC values derived was 0.000169 mg/L (UF 1000).

## Discussion

### Acute toxicity and ecological risk assessment of 4,4’-dihydroxybenzophenone

According to the result of acute toxicity test for 4,4’-dihydroxybenzophenone, the 96 h-EC_50_ on *C*. *vulgaris* was 183.60 mg/L, which showed a low-level toxicity, and the 48 h-LC_50_ of *D*. *magna* was 12.50 mg/L, which showed a medium-level toxicity. The sensitivity of *D*. *magna* was greater than that of *C*. *vulgaris*. No toxicity data of 4,4’-dihydroxybenzophenone has been reported and registered in any dominant databases, like USEPA Aquatic Toxicity Database (ECOTOX), USEPA Fathead Minnow Toxicity Database (EPAFHM) or ECETOC Aquatic Toxicity Database of European Industry Association et al., indicated that there are very few toxicity studies about this chemical.

The PNEC value of 4,4’-dihydroxybenzophenone derived from the present was 0.0125 mg/L. However, no environmental concentration of 4,4’-dihydroxybenzophenone has been reported yet, so it is still difficult to evaluate the ecological risk. It is reported that 4,4’-dihydroxybenzophenone showed the second strongest estrogenic activity in human breast cancer cell line MCF-7, compared with the strongest 2,4,4’-trihydroxybenzophenone [20]. Considering the similarity in chemical structure of the two chemicals, we could speculate that 4,4’-dihydroxybenzophenone might have the similar estrogenic activity. It is still worth attention. More work in the future on investigating its concentrations in natural environment, its acute and chronic toxicity as well as its toxicity mechanism are required.

### Acute toxicity and ecological risk assessment of 2,4,4’-trihydroxybenzophenone

According to the individual acute toxicity results of 2,4,4’-trihydroxybenzophenone, the 96 h-EC_50_ of *C*. *vulgaris* was 3.50 mg/L and the 48 h-LC_50_ of *D*. *magna* was 3.74 mg/L, which showed that the toxicity of 2,4,4’-trihydroxybenzophenone was high-level to both species, and the sensitivity of the two was similar. The PNEC value of 2,4,4’-trihydroxybenzophenone derived from AF method was 0.00350 mg/L. No toxicity data of 4,4’-dihydroxybenzophenone has been reported and registered in any dominant databases yet, indicating that there are very few toxicity studies available on the substance.

It is reported that the 4-hydroxyl group is presumably necessary for binding with the estrogen receptor, and benzophenones hydroxylated at the 2-, 4- and 4’-positions (as 2,4,4’-trihydroxybenzophenone is) showed the highest estrogen activity [20]. Kawamura reported the similar results that 2,4,4’-trihydroxybenzophenone showed the strongest estrogenic activity among all benzophenone and 19 hydroxylated derivatives, comparable to bisphenol A, due to the hydroxyl group at its 4-position [4]. It is also found that one more hydroxyl group at the 2-position can add to the estrogenic activity of 4-hydroxylated benzophenones [1]. 2,4,4’-trihydroxybenzophenone has also been reported to have antiandrogenic effect other than estrogen activity [20]. Although no environmental concentrations of 2,4,4’-trihydroxybenzophenone have been reported, its use and production, as well as the acute toxicity and estrogenic effects it exhibited, can be expected to have adverse effects on the ecosystem and should be given adequate attention. Currently, the researches on 2,4,4’-trihydroxybenzophenone are scarce, and more work needs to be done on its ambient concentrations and its toxic effect in living organisms.

### Acute toxicity and ecological risk assessment of 4-MBC

According to the acute toxicity result of 4-MBC, the 96 h-EC_50_ of *C*. *vulgaris* was 0.16874 mg/L, and the 48 h-LC_50_ of *D*. *magna* was 0.54445 mg/L. The toxicity of 4-MBC to both species was very high-level, and the sensitivity of *C*. *vulgaris* appeared to be greater than *D*. *magna*. Few toxicity data have been reported, indicating that 4-MBC has not been sufficiently studied.

According to the reported studies, 4-MBC can imitate estrogen in living organisms and cause endocrine disruption. It can trigger malignant proliferation of mammary cells and induce protein differentiation, further altering the physiological development of the organism. It is reported that the ambient concentration of 4-MBC is low and its potential of causing ecological risk is nearly negligible [21]. However, Poiger found that 4-MBC functions differently in different aquatic environment. Fish in rivers are more likely to be affected than fish in lakes, for instance, and the bioaccumulation of 4-MBC is notable [22]. Poiger also assessed effects of 4-MBC of different structures on fish, revealing that 4-MBC of transitive structure are more prevalent in the environment. This can be caused by the process of biodegradation in wastewater treatment plants. In conclusion, as the most toxic substance among all tested, 4-MBC is still worth our attention.

### Toxicity of UV filters

According to our previous researches and this study, toxicity results of 7 benzophenone-type substances and 4-MBC were compared (Table 4). As showed in Table 4, *D*. *magna* and *B*. *rerio* were generally more sensitive than *C*. *vulgaris* to the tested chemicals, indicating that fish and Daphnia can be more affected than algae in aquatic environment polluted by UV filters. Taking bioaccumulation effects into account, the hazardous effects on higher trophic level organisms are of greater concern. The toxicity of these chemicals to C. vulgaris was in the following order:

BP‐4<BP‐2<4,4’‐dihydroxybenzophenone<BP‐1<BP<2,4,4’‐trihydroxybenzophenone<BP‐3<4‐MBC.

**Table 4:** Toxicity results of UV filters.

	96 h-EC_50_ of *C*. *vulgaris (mg/L)*	48 h-LC_50_ of *D*. *magna (mg/L)*	96 h-LC_50_ of *B*. *rerio* (mg/L)	PNEC value (mg/L)	Source
BP	6.86	7.63	14.73	0.003	[16]
	(high-level)	(high-level)	(medium-level)		
BP-1	29.70	17.23	7.14	0.071	unpublished data
	(medium-level)	(medium-level)	(high-level)		
BP-2	190.67	52.81	50.80	0.51	unpublished data
	(low-level)	(medium-level)	(medium-level)		
BP-3	2.98	1.09	3.89	0.013	[15]
	(high-level)	(high-level)	(high-level)		
BP-4	201.00	47.47	633.00	0.47	[15]
	(low-level)	(medium-level)	(low-level)		
4,4’-dihydroxybenzophenone	183.60	12.50		0.0125	this study
	(low-level)	(medium-level)			
2,4,4’-trihydroxybenzophenone	3.50	3.74		0.00350	this study
	(high-level)	(high-level)			
4-MBC	0.16874	0.54445		0.000169	this study
	(very high-level)	(very high-level)			

The toxicity of these chemicals to *D*. *magna* was in the following order:

BP‐2<BP‐4<BP‐1<4,4’‐dihydroxybenzophenone<BP<2,4,4’‐trihydroxybenzophenone<BP‐3<4‐MBC.

The toxicity of these chemicals to B. rerio was in the following order:

BP‐4<BP‐2<BP<BP‐1<BP‐3.

4-MBC, BP-3 and 2,4,4’-trihydroxybenzophenone showed the highest toxicity level among all tested chemicals, and the toxicity of BP-2 and BP-4 was relatively low. Though the environmental concentration data are still rare, the endocrine disruption effect UV filters showed both *in vivo* and *in vitro* and their bioaccumulation potential make their environmental impacts non-ignorable.

### Toxicity assessment of mixing effects to *C*. *vulgaris*

The results of acute toxicity test for the mixtures were listed in Table 5. It is notable that 4,4’-dihydroxybenzophenone and 2,4,4’-trihydroxybenzophenone have similar molecular structures. Both of them contain hydroxyl and benzophenone groups, the only difference is that 2,4,4’-trihydroxybenzophenone has one more hydroxyl group than 4,4’-dihydroxybenzophenone at its 2-position. The competition they pose in the active parts of the cell surface and the metabolic system due to their structural similarity may be responsible for their antagonistic effect. Currently, researches on toxic mechanism of 4,4’-dihydroxybenzophenone, 2,4,4’-trihydroxybenzophenone and 4-MBC were barely sufficient. To precisely interpret the reason why they exhibit such effect as mixtures, more work needs to be done.

**Table 5:** Toxicity results of mixtures to *C*. *vulgaris*.

Components of Mixture	Toxicity to *C*. *vulgaris*
4,4’-dihydroxybenzophenone+ 2,4,4’-trihydroxybenzophenone	Antagonistic
4,4’-dihydroxybenzophenone + 4-MBC	Addictive
2,4,4’-trihydroxybenzophenone+ 4-MBC	Antagonistic
4,4’-dihydroxybenzophenone+2,4,4’-trihydroxybenzophenone + 4-MBC	Antagonistic

Based on the fact that UV filters are generally used in combination and exist in aquatic environment [2], emphasis should be placed on the study of mixing toxicity. The results of this study reveal the mixed toxicity of 4,4’-dihydroxybenzophenone, 2,4,4’-trihydroxybenzophenone and 4-MBC to *C*. *vulgaris*, which is important for understanding the harmful effects of these three chemicals in the aquatic environment.

## Conclusions

1. For 4,4’-dihydroxybenzophenone, the 96 h-EC_50_ value on *C*. *vulgaris* was 183.60 mg/L, the 48 h-LC_50_ value on *D*. *magna* was 12.50 mg/L. For 2,4,4’-trihydroxybenzophenone, the 96 h-EC_50_ value on *C*. *vulgaris* was 3.50 mg/L, the 48 h-EC_50_ on *D*. *magna* was 3.74 mg/L. For 4-MBC, the 96 h-EC_50_ value on *C*. *vulgaris* was 0.16874 mg/L, the 48 h-EC_50_ on *D*. *magna* was 0.54445 mg/L.
  According to the acute toxicity classification criteria, the levels of toxicity of 4,4’-dihydroxybenzophenone on *C*. *vulgaris* and *D*. *magna* were low and medium, respectively. The levels of toxicity of 2,4,4’-trihydroxybenzophenone on *C*. *vulgaris* and *D*. *magna* were high. The levels of toxicity of 4-MBC on *C*. *vulgaris* and *D*. *magna* were very high.
2. In the mixture toxicity test to *C*. *vulgaris*, the mixture of 4,4’-dihydroxybenzophenone and 2,4,4’-trihydroxybenzophenone showed antagonistic effect, while the mixture of 4,4’-dihydroxybenzophenone and 4-MBC showed addictive effect. The mixture of 2,4,4’-trihydroxybenzophenone and 4-MBC showed antagonistic effect, and the mixture of 4,4’-dihydroxybenzophenone, 2,4,4’-trihydroxybenzophenone and 4-MBC showed antagonistic effect.
3. By AF method, The PNEC values of 4,4’-dihydroxybenzophenone, 2,4,4’-trihydroxybenzophenone and 4-MBC were 0.0125, 0.00350 and 0.000169 mg/L, respectively. Due to lack of environmental concentrations data, their ecological risk can’t be determined yet.
4. In future studies, more work need to be done on environmental concentration, toxicity mechanism and toxicity testing to further assess the ecological risk of UV filters.

## Supporting information

S1 File Data and results. (XLSX)

## References

[1] Kawamura Y, Ogawa Y, Nishimura T, Kikuchi Y, Nishikawa J, Nishihara T, et al. Estrogenic activities of UV stabilizers used in food contact plastics and benzophenone derivatives tested by the yeast two-hybrid assay. J Health Sci. 2003;49(3):205–12. WOS:000183219700005.

[2] Kim S, Choi K. Occurrences, toxicities, and ecological risks of benzophenone-3, a common component of organic sunscreen products: A mini-review. Environ Int. 2014;70:143–57. 10.1016/j.envint.2014.05.015 WOS:000339693200016. 24934855

[3] Liu Q, Chen ZB, Wei DB, Du YG. Acute toxicity formation potential of benzophenone-type UV filters in chlorination disinfection process. J Environ Sci. 2014;26(2):440–7. 10.1016/s1001-0742(13)60411-8 WOS:000331781900025. 25076536

[4] Kawamura Y, Mutsuga M, Kato T, Iida M, Tanamoto K. Estrogenic and anti-androgenic activities of benzophenones in human estrogen and androgen receptor mediated mammalian reporter gene assays. J Health Sci. 2005;51(1):48–54. 10.1248/jhs.51.48 WOS:000226988700007.

[5] Peck AMJA, chemistry b. Analytical methods for the determination of persistent ingredients of personal care products in environmental matrices. 2006;386(4):907–39. 10.1007/s00216-006-0728-3 17047946

[6] Wu J-W, Chen H-C, Ding W-H. Ultrasound-assisted dispersive liquid–liquid microextraction plus simultaneous silylation for rapid determination of salicylate and benzophenone-type ultraviolet filters in aqueous samples. Journal of Chromatography A. 2013;1302:20–7. 10.1016/j.chroma.2013.06.017 23831000

[7] Kameda Y, Kimura K, Miyazaki M. Occurrence and profiles of organic sun-blocking agents in surface waters and sediments in Japanese rivers and lakes. Environmental Pollution. 2011;159(6):1570–6. 10.1016/j.envpol.2011.02.055 WOS:000290839900014. 21429641

[8] Balmer ME, Buser HR, Muller MD, Poiger T. Occurrence of some organic UV filters in wastewater, in surface waters, and in fish from Swiss lakes. Environ Sci Technol. 2005;39(4):953–62. 10.1021/es040055r WOS:000227001700008. 15773466

[9] Nguyen KTN, Scapolla C, Di Carro M, Magi E. Rapid and selective determination of UV filters in seawater by liquid chromatography-tandem mass spectrometry combined with stir bar sorptive extraction. Talanta. 2011;85(5):2375–84. 10.1016/j.talanta.2011.07.085 WOS:000296115600017. 21962656

[10] Zhang Z, Ren N, Li Y-F, Kunisue T, Gao D, Kannan K. Determination of Benzotriazole and Benzophenone UV Filters in Sediment and Sewage Sludge. Environ Sci Technol. 2011;45(9):3909–16. 10.1021/es2004057 21480589

[11] Daughton CG, Ternes TAJEhp. Pharmaceuticals and personal care products in the environment: agents of subtle change? 1999;107(suppl 6):907–38. 10.1289/ehp.99107s6907 10592150 PMC1566206

[12] Fent K, Zenker A, Rapp M. Widespread occurrence of estrogenic UV-filters in aquatic ecosystems in Switzerland. Environmental Pollution. 2010;158(5):1817–24. 10.1016/j.envpol.2009.11.005 WOS:000277726500092. 20004505

[13] Fent K, Kunz PY, Zenker A, Rapp MJMer. A tentative environmental risk assessment of the UV-filters 3-(4-methylbenzylidene-camphor), 2-ethyl-hexyl-4-trimethoxycinnamate, benzophenone-3, benzophenone-4 and 3-benzylidene camphor. 2010;69:S4–S6. 10.1016/j.marenvres.2009.10.010 19910045

[14] Fong HC, Ho JC, Cheung AH, Lai K, William KJC. Developmental toxicity of the common UV filter, benophenone-2, in zebrafish embryos. 2016;164:413–20. 10.1016/j.chemosphere.2016.08.073 27599007

[15] Du Y, Wang WQ, Pei ZT, Ahmad F, Xu RR, Zhang YM, et al. Acute Toxicity and Ecological Risk Assessment of Benzophenone-3 (BP-3) and Benzophenone-4 (BP-4) in Ultraviolet (UV)-Filters. International Journal of Environmental Research and Public Health. 2017;14(11). 10.3390/ijerph14111414 WOS:000416545200130. 29156601 PMC5708053

[16] Sun H-Q, Du Y, Zhang Z-Y, Jiang W-J, Guo Y-M, Lu X-W, et al. Acute toxicity and ecological risk assessment of benzophenone and N, N-Diethyl-3 Methylbenzamide in personal care products. 2016;13(9):925. 10.3390/ijerph13090925 PMC5036758 27657095

[17] Health Mo. Chemicals-Alga Growth Inhibition Test. People’s Republic of China: Standard Administration of China; 2008.

[18] Health Mo. Method for Acute Toxicity Test of Daphnia Magna Straus. People’s Republic of China: Standardization Administration of China; 2012.

[19] Wei XW F.; Qi W. Monitoring Analysis Method of Water and Wastewater. 4th ed. Beijing, China: Ministry of Environmental Protection of People’s Republic of China; 2013. 767–81 p.

[20] Suzuki T, Kitamura S, Khota R, Sugihara K, Fujimoto N, Ohta SJT, et al. Estrogenic and antiandrogenic activities of 17 benzophenone derivatives used as UV stabilizers and sunscreens. 2005;203(1):9–17. 10.1016/j.taap.2004.07.005 15694459

[21] Sang Z, Leung KS-Y. Environmental occurrence and ecological risk assessment of organic UV filters in marine organisms from Hong Kong coastal waters. Science of The Total Environment. 2016;566–567:489–98. 10.1016/j.scitotenv.2016.05.120 27235899

[22] Poiger T, Buser H-R, Balmer ME, Bergqvist P-A, Müller MDJC. Occurrence of UV filter compounds from sunscreens in surface waters: regional mass balance in two Swiss lakes. 2004;55(7):951–63. 10.1016/j.chemosphere.2004.01.012 15051365

